# P-1726. Mucormycosis coinfection by plasma Mucorales PCR in patients with proven aspergillosis

**DOI:** 10.1093/ofid/ofaf695.1897

**Published:** 2026-01-11

**Authors:** Hyeon Mu Jang, Ji Yeon Kim, Hyeonji Seo, Sung-Han Kim

**Affiliations:** Asan medical center, Seoul, Seoul-t'ukpyolsi, Republic of Korea; Asan medical center, Seoul, Seoul-t'ukpyolsi, Republic of Korea; Asan Medical Center, Seoul, Seoul-t'ukpyolsi, Republic of Korea; Asan medical center, Seoul, Seoul-t'ukpyolsi, Republic of Korea

## Abstract

**Background:**

Diagnosis of mucormycosis is challenging due to lack of antigen testing and low sensitivity of fungal culture, especially when biopsy is not feasible. There are limited data on coinfection of Aspergillus and Mucorales, although identifying coinfection is important for management. Thus, we systematically investigated mucormycosis coinfection in proven or probable aspergillosis, using plasma Mucorales PCR.

**Table.**
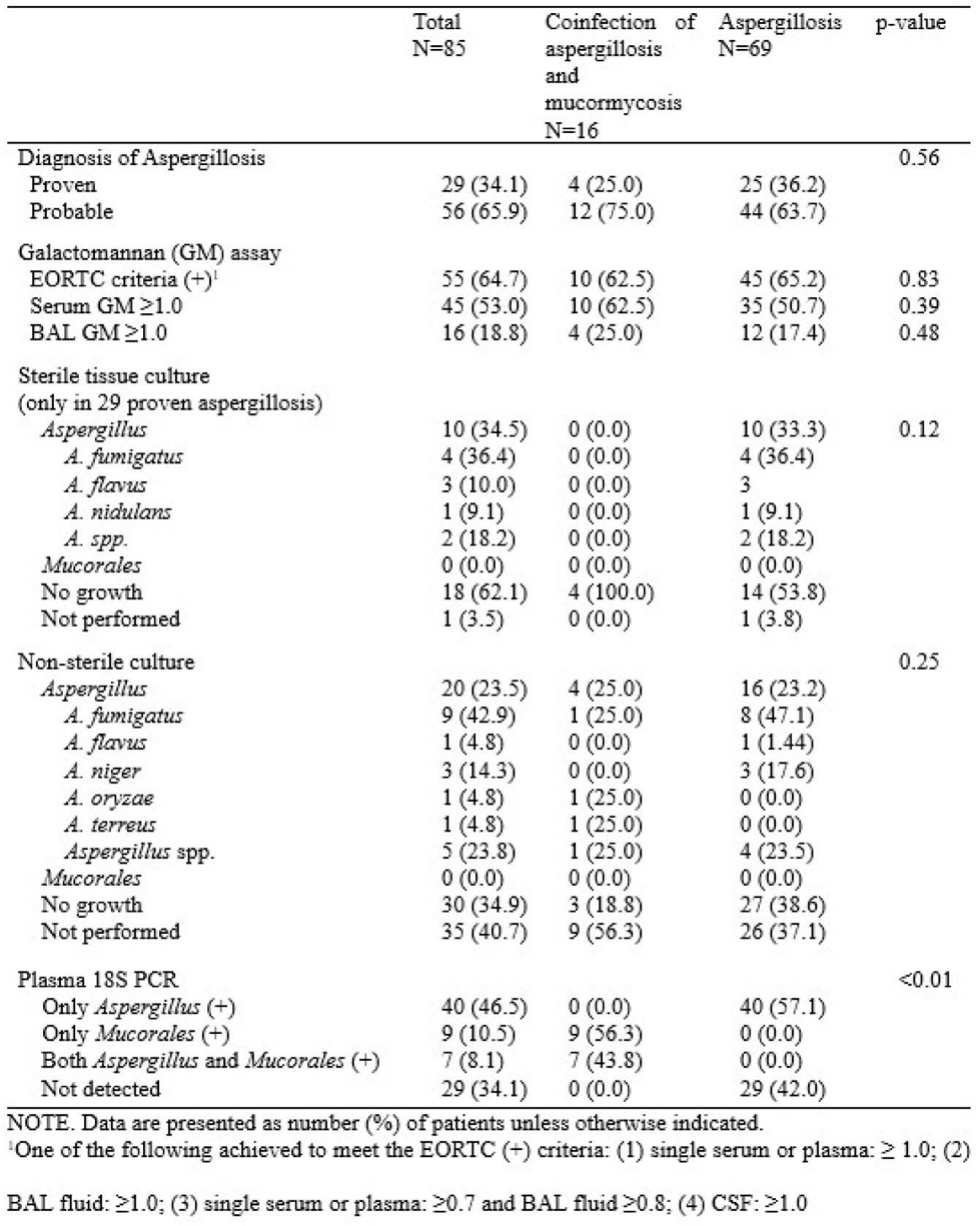
Diagnostic characteristics of the study participants

**Methods:**

The medical records of adult patients with proven and probable aspergillosis were retrospectively reviewed at a tertiary hospital during Jan 2017 to Apr l 2020 and Jun 2023- Feb 2025. The fungal culture results were reviewed. The plasma 18S PCR was performed to detect *Aspergillus*- and *Mucorales*-specific DNA.

**Results:**

A total of 85 patients with proven 29 and probable 56 aspergillosis were analyzed. No Mucorales was found in sterile and non-sterile cultures. In 29 patients with proven aspergillosis, a positive *Mucorales*-specific PCR result was obtained for 2 patients (6.9%) while positive *Aspergillus*- and *Mucorales*-specific PCR results were obtained for 2 patients (6.9%). In proven aspergillosis, 13.8% (4/29) showed evidence of mucormycosis coinfection. In 56 patients with probable aspergillosis, a positive *Mucorales*-specific PCR result was obtained for 7 patients (12.5%) while positive *Aspergillus*- and *Mucorales*-specific PCR results were obtained for 5 patients (8.9%). In probable aspergillosis, 21.4% (12/56) showed evidence of mucormycosis coinfection. Overall, 16 (18.8%) of the 85 patients with proven or probable aspergillosis showed evidence of mucormycosis coinfection. In hospital mortality did not differ between patients with mucormycosis coinfection who was treated with voriconazole and those with aspergillosis alone with voriconazole (38.5% [5/13] vs 29.4% [15/51]; p 0.52).

**Conclusion:**

About 15-20% of patients with proven or probable aspergillosis had molecular evidence of mucormycosis coinfection by plasma Mucorales PCR. Further studies are needed.

**Disclosures:**

All Authors: No reported disclosures

